# Logistic regression analysis on the determinants of stillbirth in Ethiopia

**DOI:** 10.1186/s40748-016-0038-5

**Published:** 2016-09-15

**Authors:** Kidanemariam Alem Berhie, Habtamu Gebremariam Gebresilassie

**Affiliations:** 1 Department of Statistics, College of Natural and Computational Sciences, University of Gondar, Gondar, Ethiopia; 2 Department of Statistics, College of Natural and Computational Sciences, Dilla University, Dilla, Ethiopia

**Keywords:** Stillbirth, Antenatal care visit, Logistic regression, Ethiopia

## Abstract

**Background:**

Stillbirth is often defined as fetal death after 24 weeks of gestation, but a fetus greater than any combination of 16, 20, 22, 24, or 28 weeks gestational age and 350 g, 400 g, 500 g, or 1000 g birth weight may be considered stillborn depending on local law. Once the fetus has died, the mother may or may not have contractions and undergo childbirth or in some cases, a Caesarean section. Most stillbirths occur in full-term pregnancies.

**Methods:**

This study has intended to model determinants of experiencing stillbirth among women in child bearing age group of Ethiopia using the Ethiopian demographic and health Survey data (EDHS, 2011). First, the bivariate chi-square test of association was fitted to the data and significant variables were considered for further investigation binary logistic regression models were fitted.

**Results:**

This study revealed that the rate of experiencing stillbirth among women of child bearing age was about 25.5 per 1000 deliveries in Ethiopia. From binary logistic regression, region of residence, maternal age, place of residence, education level, parity, antenatal care utilization, place of delivery, body mass index (BMI) and anemia level were found to be significantly associated with experiencing stillbirth.

**Conclusions:**

Researchers should use multilevel models than traditional regression methods when their data structure is hierarchical as like in Ethiopian Demographic and Health Survey data.

## Background

Stillbirth is often defined as fetal death after 24 weeks of gestation [1], but a fetus greater than any combination of 16, 20, 22, 24, or 28 weeks gestational age and 350 g, 400 g, 500 g, or 1000 g birth weight may be considered stillborn depending on local law [2]. Once the fetus has died, the mother may or may not have contractions and undergo childbirth or in some cases, a Caesarean section. Most stillbirths occur in full-term pregnancies. The cause is often unknown.

The 2011 Lancet Stillbirths Series reviewed the global status of stillbirths and presented the case for a triple return on investment in stillbirth prevention that also prevents newborn and maternal deaths. That Series received widespread media attention and an unprecedented response [3]. However, despite progress this new Series shows that more must be done to integrate stillbirth prevention within global and national agendas for high quality health care for women, adolescents, and babies. This message resonates with other Lancet Series, notably on maternal health, early child development, and Every Newborn.

Most of the world’s 2.6 million stillbirths each year occur in low-income and middle-income countries (98 %), with three quarters in sub-Saharan Africa and south Asia. About 60 % occur in rural areas and more than half in conflict and emergency zones, affecting the families most underserved by health-care systems. Stillbirths are often not registered systematically in many low-income countries. This leads to underestimation of stillbirths in these countries, in which 98 % of all stillbirths occur. Reliable registrations exist only in countries with minor number of deaths. India, Pakistan, Nigeria, China, Bangladesh, Democratic Republic of the Congo, Ethiopia, Indonesia, Tanzania and Afghanistan are ten countries that account for two-thirds of all third trimester stillbirths. Ethiopia is ranked number seven out of these ten [4].

If all causes of stillbirth are taken together, the new estimates would place stillbirths fifth on the list of causes of deaths (COD) worldwide, two-thirds of stillbirths happen in rural areas, where skilled birth attendants, in particular midwives and physicians, are not always available for essential care during childbirth and for obstetric emergencies, including caesarean sections [5].

Over 98 % of the perinatal deaths occur in low and middle-income countries (LMIC), with more than 70 % occurring in community settings, often the home, far from vital registration/formal health systems [6–8], under-reporting of stillbirths is a huge problem, and reliable data about rates and causes are difficult to obtain. Hospital stillbirth data are often subject to substantial bias and the ability to generalize from these data is unknown. Nevertheless, of the stillbirths occur worldwide yearly, the vast majority in developing countries, with rates in many developing countries ten-fold higher than elsewhere. Prolonged and obstructed labor, preeclampsia and various infections, all without adequate treatment, account for the majority of stillbirths [9].

In Ethiopia, the world health statistics 2013 revealed a stillbirth rate of 26/1000 deliveries which is third highest in the east African countries next to Djibouti and Somalia (with stillbirth rates of 34 & 30 per 1000 births, respectively) [10] and seventh among the ten countries that account for two-thirds of all third trimester stillbirths in the world [11], a study reported that the prevalence of stillbirth is 19/1000 births [12]. A study done at Tikur Anbessa Hospital has shown a stillbirth rate of 53.3/1000 births and contributed to 77.2 % of gross perinatal mortality [13]. The Ethiopian Demographic and Health Survey (DHS) 2005 data indicated that the still birth rate is 1.8 % [14]. The Addis Ababa city administration health bureau 2005/06 annual activity report revealed that the rate of stillbirth is 2.5 % [15]. A study done, recently, on prenatal outcomes in Addis Ababa in 2010, also indicates that the rate of stillbirth is 3.1 % [16].

Most of the mothers and grandmothers associated the causes of stillbirth and neonatal death with malevolent spirits. As one Oromiya grandmother observed, “Families lose their new born because of an evil spirit”. (Wukabi is a type of malevolent spirit that, when offended, will attack the beholder or his/her family) [17].

Goal by 2020 [18]; For countries with a current stillbirth rate of more than 5 per 1000 births, the goal by 2020 is to reduce their stillbirth rates by at least 50 % from the 2008 rates. For countries with a current stillbirth rate of less than 5 per 1000 births, the goal by 2020 is to eliminate all preventable stillbirths and close equity gaps [19].

Therefore, this study attempts to investigate the major socio-economic, demographic, medical, behavioral and environmental factors of stillbirth in Ethiopia so that the SDG and goal by 2020 for stillbirth will be met.

### Objective of the study

#### General objective

The general objective of this study is to assess the determinants of stillbirth in Ethiopia using Ethiopian Demographic and Health Survey 2011 data.

#### Specific objectives

- ➢ To assess socio-economic, demographic, and medical factors associated with stillbirth.
- ➢ To identify factors that may explain the variation of rate of stillbirth

### Data and methodology

#### Source of data

This study has used the 2011 Ethiopia Demographic and Health Survey (2011, EDHS) [20]. The 2011 EDHS was conducted under the aegis of the ministry of health and was implemented by the Central Statistical Agency and partner organizations from September 2010 through June 2011 with a nationally representative sample of nearly 18,500 households. All women age 15–49 and all men age 15–59 in these households were eligible for individual interview.

The sample for the 2011 EDHS was designed to provide population and health indicators at the national and regional levels. The sample design allowed for specific indicators, such as stillbirth experience, to be calculated for each of Ethiopia’s eleven geographic/administrative regions: nine regional states (Tigray, Afar, Amhara, Oromiya, Somali, Benishangul-Gumuz, SNNPR, Gambela and Harari) and two city administrations (Addis Ababa and Dire-Dawa). The sampling frame used for the 2011 EDHS was the Population and Housing Census conducted by the Central Statistical Agency (CSA) in 2007 (2007 PHC). The 2011 EDHS sample was selected using a stratified, two-stage cluster design, and EAs were the sampling units for the first stage sampling. The 2011 EDHS sample included 624 EAs, 187 in urban areas and 437 in rural areas.

Households comprised the second stage of sampling. A complete listing of households was carried out in each of the 624 selected EAs from September 2010 through January 2011. Maps were drawn for each of the clusters and all private households were listed. The listing excluded institutional living arrangements (e.g., army barracks, hospitals, police camps, and boarding schools). A representative sample of 17,817 households was selected for the 2011 EDHS survey. Because the sample is not self-weighting at the national level, all data in this report have been weighted unless otherwise specified. Sixteen thousand five hundred fifteen women aged 15–49 are interviewed, 12,560 women after adjusting for the missing data have been taken for the analysis.

### Variables of the study

Variables considered in this study were selected based on literatures which have been conducted at the global level. Potential determinant factors expected to be correlated with stillbirth among mothers of child bearing age are included as variables of the study. Variables considered in this study are categorized into dependent and explanatory or predictor variables.

### Dependent variable

The 2011 EDHS asked women to report any pregnancy loss that occurred in the five years preceding the survey. For each pregnancy that did not end in a live birth, the duration of the pregnancy was recorded. Pregnancy losses occurring after seven completed months of gestation are defined as stillbirths. The response variable of this study is the occurrence of stillbirth among mothers of child bearing age.

The response variable for the i^th^ mother (15–49) is represented by a random variable Y_i_ with two possible values coded as 1 and 0. So, the response variable of the i^th^ mother Y_i_ was measured as a dichotomous variable with possible values Y_i_ = 1, if i^th^ mother had experienced stillbirth and Y_i_ = 0 otherwise.

## Methods

In this study binary logistic regressions were employed to identify determinant risk factors of stillbirth and to determine the prevalence of stillbirth in Ethiopia. The response variable of the study is experiencing stillbirth prior to the survey. We analyzed using single level binary logistic regressions by assuming the occurrence of stillbirth is independent among mothers of child bearing age.

## Results

### Descriptive statistics

We analyzed data from women of child bearing age from the Ethiopian Demographic and Health Survey 2011 sample. The initial population consisted of 16,515 women of child bearing age. Out of this 12,560 (76 %) of women with complete information were selected and studied in the analysis. From the sampled women, the proportion of experiencing stillbirth was about 2.55 % (25.5 per 1000) in Ethiopia.

The analysis is carried out in two parts. In the first part, we present the bivariate analysis with its chi-square test of association and then selecting the significant variables, we analyze the data using ordinary logistic regression, for both the analyses we used SPSS 20 software.

### Result of bivariate analysis

The sample distribution of region of residence, maternal age, place of residence, Education level, wealth index, Parity (Total children ever born), Antenatal care utilization, Place of Delivery (home or health center), Mode of Delivery (normal or by caesarean section), Body mass index (BMI), marital status, whether they had any STI or not, whether they smoke cigarettes or not, Anemia level (Anemic or not), whether they have job or not and frequency of consuming alcoholic drink are presented in Table 1.

**Table 1:** Distribution of factors analyzed with experiencing stillbirth among women of child bearing age in Ethiopia (EDHS, 2011)

Variables	Levels	N	N%	Experienced Stillbirth %		d.f	Chi-square	*p*-value
				No	Yes			
Region	Addis Ababa	1167	9.3	98.46	1.54	10	53.262	< .001
	Tigray	1368	10.9	96.27	3.73			
	Affar	1034	8.2	98.26	1.74			
	Amhara	1554	12.4	98.13	1.87			
	Oromiya	1705	13.6	96.48	3.52			
	Somali	703	5.6	94.74	5.26			
	Benishangul-Gumuz	975	7.8	97.23	2.77			
	SNNP	1640	13.1	97.62	2.38			
	Gambela	872	6.9	98.51	1.49			
	Harari	742	5.9	98.38	1.62			
	Dire Dawa	800	6.4	98.00	2.00			
Maternal age	15–24	6363	50.7	98.88	1.12	2	144.68	< .001
	25–34	4179	33.3	96.84	3.16			
	35+	2018	16.1	94.20	5.80			
Place of residence	Rural	8924	71.1	95.38	4.62	1	8.407	.004
	Urban	3636	28.9	97.63	2.37			
Education Level	No education	5955	47.4	96.47	3.53	2	43.691	< .001
	Primary	4835	38.5	98.32	1.68			
	Secondary & Higher	1770	14.1	98.36	1.64			
Wealth Index	Poor	4924	39.2	97.42	2.58	2	1.439	.487
	Middle	1784	14.2	97.09	2.91			
	Rich	5852	46.6	97.59	2.41			
Parity (Total children ever born)	Nulliparous	5169	41.2	98.99	1.01	1	84.097	< .001
	Multiparas	7391	58.8	96.37	3.63			
Antenatal care utilization	No antenatal visits	4101	32.7	93.34	6.66	2	97.417	< .001
	Visited at least once	3290	26.2	96.41	3.59			
	Never given birth	5169	41.2	98.99	1.01			
Place of Delivery	HOME	6166	49.1	92.40	7.60	2	138.53	< .001
	Health center	1225	9.8	96.24	3.76			
	Never given birth	5169	41.2	98.99	1.01			
Mode of Delivery	Normal	7169	57.1	94.42	5.58	2	85.725	< .001
	Caesarean section	222	1.8	96.05	3.95			
	Never given birth	5169	41.2	98.99	1.01			
Body mass index (BMI)	Thin	3541	28.2	95.83	4.17	2	134.32	< .001
	Normal	8248	65.7	97.37	2.63			
	Overweight/Obese	771	6.1	93.63	6.37			
Marital status	Never Married	4017	32.0	97.13	2.87	2	4.067	.131
	Married/Living with partner	7640	60.8	96.32	3.68			
	Divorced/separated/Widowed	903	7.2	96.46	3.54			
Had any STI	No	12498	99.5	97.46	2.54	1	0.115	.471
	Yes	62	.5	96.77	3.23			
Smokes cigarettes	No	12513	99.6	97.44	2.56	1	1.233	.297
	Yes	47	.4	100.0	0.00			
Anemia level	Not anemic	10010	79.7	97.65	2.35	1	11.953	< .001
	Anemic	2550	20.3	94.67	5.33			
Has job	No	12340	98.2	97.51	2.49	1	6.190	.013
	Yes	220	1.8	96.09	3.91			
Alcoholic drink	Never	8454	67.3	97.60	2.40	3	3.669	.299
	Sometimes	2945	23.4	97.32	2.68			
	Usually	683	5.4	96.93	3.07			
	Always	478	3.8	96.44	3.56			

Among the factors, region of residence, maternal age, place of residence, education level, parity, antenatal care utilization, place of delivery, mode of delivery, body mass index (BMI), and anemia level were found to have a significant association with experiencing stillbirth at 1 % level of significance (*p*-values less than 0.01), while having job was significant at the 5 % level of significance (*p*-values less than 0.05).

Experiencing stillbirth has varied from one region to the other. The result in Table 1 shows that region of residence is significantly associated with experiencing stillbirth (*p* < 0.001). Somali region had the highest (5.26 %) percentage of experiencing stillbirth followed by Tigray region (3.73 %). Gambela and Addis Ababa had the lowest percentages (1.49 %, 1.54 %) respectively, for experiencing stillbirth in Ethiopia.

Of the 12,560 women with complete information, 50.7 % were 15–24 years old, 33.3 % were 25–34 years old and the rest (16.1 %) were 35 or above. Maternal age was significantly associated with experiencing stillbirth and it was found that mothers with higher age were found to be with higher probability to experience stillbirth. Place of residence was also significantly associated with experiencing stillbirth and of the 71.1 % rural area resident women 4.62 % had experienced stillbirth and only 2.37 % urban area residents had experienced stillbirth.

Education level is also associated with experiencing stillbirth. 47.4 % of the women were with no educational achievement and, of this, those who had experienced stillbirth were 3.53 % as compared to that of those who completed their primary education (1.68) and to that of those who completed secondary or higher education level (1.64). 41.2 % of the women were nulliparous, having no child, with 1.01 % proportion of experiencing stillbirth as compared to 3.63 % proportion of experiencing stillbirth among multiparas.

Table 1 displayed also that among the women of child bearing age 26.2 % has made their antenatal care visit at least once during their pregnancy times and 3.59 % of these had experienced stillbirth, which is less than that of those (32.7 %) who made no antenatal care visit during their pregnancies which was 6.66 %. 41.2 % of the women had no child and among the women who delivered a child, 49.1 % had delivered at home in which 7.6 % were stillbirth, only 9.8 % had delivered at any health center in which 3.76 % were stillbirth, 57.1 % had delivered normally in which 5.58 % were stillbirth and 1.8 % of the women delivered with caesarean section in which 3.95 % of those delivered with caesarean section had given to stillbirth.

Body mass index was found to be another significantly associated with experiencing stillbirth. The result indicates that 28.2 % of the women were thin (body mass index (BMI) < 18.5), 65.7 % were normal (weight) (BMI 18.5–24.9) and 6.1 % were overweight or obese (BMI ≥ 25). The proportion of experiencing stillbirth among women who are thin, normal and overweight were 4.17 %, 2.63 % and 6.37 % respectively. 79.7 % of the women were not anemic and with less proportion of experiencing stillbirth than those (20.3 %) with anemia whose proportion is 5.33 %. 98.2 % of the women were having no job while 1.8 % had any job. The proportion of experiencing stillbirth among those who had no job was 2.49 % which is less than that of those who had any job (3.91 %).

### Results of binary logistic regression analysis

Multiple logistic regression models were fitted using the categorical predictor variables which were found to be significant in the bivariate analysis using enter selection (Likelihood ratio) method. The results are presented in Table 5. The result shows that nine of the predictor variables were significantly associated with experiencing stillbirth.

### Assessment of goodness of fit of the model

For categorical data, after we fit the logistic model, it is necessary to see the appropriateness, adequacy and usefulness of the fitted model. To overcome this we have several techniques. The most commonly used techniques are Pearson’s Chi-square, the likelihood ratio tests (LRT) and Hosmer and Lemeshow Goodness of fit test.

The result presented in Table 2 showed a likelihood ratio test statistic G^2^ = 277.041 which is distributed as chi-square with 11° of freedom. The tabulated value was X_0.05_^2^(11) = 19.675. Since G^2^ > X_0.05_^2^(11), we reject the null hypothesis and conclude that at least one of the predictors was significantly related with experiencing stillbirth among mothers of child bearing age.

**Table 2:** Overall model evaluation using likelihood ratio test (EDHS, 2011)

	-2 Log likelihood	Likelihood ratio (G^2^)	d.f	X_α_^2^(16)
Null model	3043.857	277.041	11	19.675
Full model	2766.816			

Based on the results in Table 3, the null hypothesis that there is no difference between the model with only a constant and the model with independent variables was rejected.

**Table 3:** Omnibus tests of model coefficients (EDHS, 2011)

	Chi-square	d.f	Sig.
Step	213.727	23	0.000
Block	213.727	23	0.000
Model	213.727	23	0.000

The Hosmer-Lemeshow goodness-of-fit test is found to be not significant (*x*^2^ = 7.424, d.f = 8, *p*-value = 0.72). Thus, we do not have an evidence to reject the null hypothesis that the model fitted the data well.

The Nagelkerke R-square was 8.0 % indicating that explanatory variables were useful in predicting experiencing stillbirth. But, it doesn’t give the meaning of variance explained as in linear regressions (33).

### Validation of predicted probabilities

The degree to which predicted probabilities agree with actual outcomes is expressed as a classification table. Classification table documents the validity of predicted probabilities. According to the classification presented in Table 4, prediction for women who had not experienced stillbirth was more accurate than that for those who had experienced stillbirth. This observation is supported by the magnitude of sensitivity (81.6 %) compared to that of specificity (100.0 %). Sensitivity measures the proportion of correctly classified events (i.e., those women who had experienced stillbirth), whereas specificity measures the proportion of correctly classified nonevents (those women who had not experienced stillbirth). The overall correct prediction was 99.5 %, an improvement over the chance level.

**Table 4:** Classification table of model with predictor variables (EDHS, 2011)

Observed		Predicted		
		Experienced stillbirth		Percentage correct
		No	Yes	
Experienced stillbirth	No	12240	0	100.0
	Yes	59	261	81.6
Overall Percentage				99.5

### Interpretation of logistic regression coefficients

A multiple logistic model was fitted to the data to test the research hypothesis regarding the relationship between the likelihood that experiencing stillbirth of woman is related with the predictor variables. Result displayed in Table 5 revealed that region of residence, maternal age, place of residence, education level, parity, antenatal care utilization, place of delivery, body mass index (BMI) and anemia level were found to be significantly associated with experiencing stillbirth.

**Table 5:** Logistic regression results of experiencing stillbirth among women, in Ethiopia

	B	S.E.	Wald	Df	Sig.	Exp(B)	95 % CI for Exp(B)	
							Lower	Upper
AGE (15–24 ref.cat)			49.686	2	.000			
25–34	1.250	.183	46.463	1	.000	3.49	2.438	4.996
35+	1.917	.333	33.140	1	.000	6.8	3.541	13.062
REGION (Addis Ababa ref.cat)			41.254	10	.000			
Tigray	-.035	.359	.009	1	.922	0.966	0.478	1.952
Affar	.851	.304	7.822	1	.005	2.342	1.291	4.250
Amhara	-.089	.357	.063	1	.803	0.915	0.454	1.842
Oromiya	.103	.328	.099	1	.754	1.108	0.583	2.108
Somali	.782	.300	6.794	1	.009	2.185	1.214	3.935
Benishangul-Gumuz	.897	.314	8.167	1	.004	2.451	1.325	4.538
SNNP	.478	.332	2.074	1	.150	1.613	0.841	3.092
Gambela	.358	.317	1.276	1	.259	1.431	0.768	2.663
Harari	-.109	.387	.079	1	.779	0.897	0.420	1.915
Dire Dawa	-.063	.389	.026	1	.871	0.939	0.438	2.013
ResidPlace (Rural ref.cat)								
Urban	-.478	.176	7.370	1	.007	0.62	0.439	0.875
EucationLevel (No Educ.ref.cat)			8.254	2	.016			
Primary	-.291	.252	1.342	1	.247	0.748	0.456	1.225
Secondary & Higher	-1.728	.239	7.230	1	.007	0.178	0.111	0.284
PARITY (Nulliparous ref.cat)								
Multipara	1.168	.362	10.385	1	.001	3.215	1.582	6.537
ANC (No ANC visit ref.cat)			5.953	1	.015			
Visited atleast once	-.729	.299	5.953	1	.015	0.482	0.268	0.867
DeliveryPlace (Home ref.cat)			.201	1	.654			
Health center	-1.417	.435	10.611	1	.001	0.242	0.103	0.569
Delivery Mode (Normal ref.cat)			1.894	1	.169			
Caesarean section	-.498	.362	1.894	1	.169	0.608	0.299	1.236
BMI (Thin ref.cat)			7.757	2	.021			
Normal	-.729	.152	4.796	1	.029	0.482	0.358	0.650
Overweight/Obese	.417	.230	1.815	1	.178	1.518	0.967	2.382
ANEMIA (Not anemic ref.cat)								
Anemic	.916	.436	4.414	1	.036	2.499	1.063	5.874
OCCUPATION (No ref.cat)								
Yes	-.492	.319	2.379	1	.123	0.611	0.327	1.143
Constant	-1.522	.532	8.188	1	.004	0.218	0.077	0.619

*ref.cat* reference category, *SNNP* South Nations Nationalities and Peoples

Experiencing stillbirth was significantly associated with geographical regions. The odds of experiencing stillbirth in Tigray, Amhara, Oromiya, SNNP, Gambela, Harari, and Dire-Dawa were not significantly different from that of experiencing stillbirth in Addis Ababa. Experiencing stillbirth in Benishangul-Gumuz was 2.451 times more likely than that in Addis Ababa city. Women who live in Afar and Somali were more likely to experience stillbirth than women who live in Addis Ababa (Table 5).

According to the model, the log of the odds of a woman to experience stillbirth was positively related to maternal age group 25–34 (*p* = 0.000) and 35+ (*p* = 0.000) when compared with age group 15–24. Indicating that the older the woman the more likely to experience stillbirth. The odds of a woman in age group (25–34) of experiencing stillbirth were 3.49 times the odds of woman with age group 15–24 and the odds of a woman in age group (35+) of experiencing stillbirth were 6.80 times that of a woman with age group 15–24. This further indicates that women of older ages are vulnerable to experiencing stillbirth.

Table 5 has also revealed that place of residence was significantly associated with experiencing stillbirth. The likelihood of experiencing stillbirth for those women residing in urban area is 0.620 times that of those women residing in rural area. Educational level was also found to be significantly associated with experiencing stillbirth. Though women having only primary education have no significant difference in experiencing stillbirth with those having no educational attainment, women with secondary and higher education were less likely (OR = 0.178) to experience stillbirth than those with no educational attainment.

Table 5 is trying to tell us that the multipara women, those having at least one child, were 3.215 times more likely vulnerable to experience stillbirth than the nulliparous women, those having no children. Women who have made antenatal care visit for at least once during their pregnancy times were less likely (OR = 0.482) to experience stillbirth than those who haven’t visited antenatal care. Women who delivered their babies at any health center were 75.8 % (0.242-1, OR = 0.242) less likely to experience stillbirth than those who preferred to deliver at home.

Experiencing stillbirth is significantly associated with the body mass index (BMI) of women. The normal weight women were found to be less likely (OR = 0.482) to experience stillbirth than those women who were thin (BMI < 18.5). Although not significant, those women who were overweight/obese (BMI ≥ 25) were more likely (1.518) to experience stillbirth than those women who were thin. Thus, normal weight women were found to be less likely to experience stillbirth than abnormal weight women. Women who were anemic are 2.499 times likely to experience stillbirth than those who were not anemic.

## Discussions

This study has intended to model determinants of experiencing stillbirth among women in child bearing age group of Ethiopia using the Ethiopian demographic and health Survey data. Accordingly, different models are fitted to the data to identify potential determinants of experiencing stillbirth among women in reproductive age group. First, the bivariate chi-square test of association was fitted to the data and significant variables were considered for further investigation in binary logistic regression model.

This study revealed that the rate of experiencing stillbirth among women of child bearing age was about 25.5 per 1000 deliveries in Ethiopia consistent with the world health statistics 2013 report which revealed a stillbirth rate of 26/1000 deliveries and was also almost consistent with a large review of data for 190 countries which estimated a stillbirth rate of 32/1000 deliveries in South Asia and Sub-Saharan Africa [4].

Among the factors, mode of delivery and occupation were found to have a significant association with experiencing stillbirth only in the bivariate analysis. And factors like education level, parity, body mass index (BMI) and anemia level were significantly associated with experiencing stillbirth also in binary logistic regression which is consistent with most of the studies in the literature [21–23].

The rate of experiencing stillbirth in Tigray, Amhara, Oromiya, SNNP, Gambela, Harari and Dire Dawa were not significantly differing from that in Addis Ababa. This might be because of most of these regions are similarly developed as Addis Ababa. Women who live in Afar, Somali and Benishangul-Gumuz regions were significantly more likely to experience stillbirth than those women living in Addis Ababa which might be because of they were disadvantaged regions in the past reigns.

This study revealed that experiencing stillbirth among women was significantly associated with the age group they are found in. Women in higher age group, especially those above 35 years, are more likely to experience stillbirth than those at lower age group. This finding was consistent with a study done using available data from 6 study sites of The Newborn Cross-Sectional Study (NCSS), component of INTERGROWTH-21st, maternal age >40 (OR: 2.52) [22]. Silver et al. [21] has reviewed researches done on five clinical sites in America stated that the stillbirth rate is increased two-fold for women 35–39 years of age, and 3- to 4-fold for women aged forty or older. While some age-associated risk is due to higher rates of maternal complications, in uncomplicated pregnancies there may be a 50% increased risk associated only with maternal age ≥ 35. For older women, stillbirth risk rises more rapidly as gestational age increases beyond 37 weeks. A prospective study done in Nigeria also revealed that 35 years and above pregnancy was important factor contributing to high stillbirth rate [24]. And almost all researchers in the literature agree in that advanced maternal age is contributing factor to high stillbirth rate.

Women’s place of residence was found to be significantly associated with experiencing stillbirth. Those women residing in rural areas were found to be more likely to experience stillbirth than those in urban areas which might be for the reason that in rural areas there is lack of a skilled attendant at delivery, lack of education, lack of full information and so on. This finding was in line with the finding of review of causes, risk factors and prevention strategies of stillbirth in developing countries [25].

Experiencing stillbirth was also significantly associated with utilization of antenatal care (ANC). Visiting antenatal care for at least once is found to decrease the probability of experiencing stillbirth. This finding has an agreement with a cross-sectional retrospective analysis of stillbirth among women delivering in University of Maiduguri teaching hospital (UMTH), east Nigeria in which lack of antenatal care visit (OR: 1.91) had increased the rate of experiencing stillbirth [26]. A research finding also revealed that lack of antenatal care had positive association with stillbirth [24]. In the binary logistic analysis done in Hawassa University Hospital, southern Ethiopia, both the crude and adjusted analysis showed that the stillbirth rate was highest among mothers who had no Antenatal Care follow up [27].

Delivering at health center rather than delivering at home brought about less probability to experience stillbirth. This finding was consistent with a prospective study entitled ’Causes of stillbirth in a community survey in Gombe State’, Nigeria [24]. This happens because when mothers deliver at home, they might not find skilled attendant and in difficult case there is no other choice like caesarean section in health centers.

## Conclusions

The purpose of this study has been to assess socio-economic, demographic, and medical factors associated with stillbirth in Ethiopia. The descriptive result showed that 25.5 per 1000 deliveries were stillbirth.

In this study single level logistic regression were used. In the single level logistic regression model, region of residence, maternal age, place of residence, education level, parity, antenatal care utilization, place of delivery, body mass index (BMI) and anemia level were found to be significantly associated with experiencing stillbirth. Women of older ages are vulnerable to experiencing stillbirth. Chi-square test of association was done to see if there is association between experiencing stillbirth and region of residence and since it revealed that region of residence was associated with experiencing stillbirth.

### Recommendations

Based on the findings of this study we forward the following recommendations to whom it my concern:

- ✓ First and for most all mothers should take care of their health condition when they become pregnant, during pregnancy and when approaching to labour. This can be made by utilizing antenatal care in health centers.
- ✓Mothers should prefer and people who are around them should advise them to give birth at health centers than delivering at home.
- ✓Those older age women, above 35 years, should be more careful for difficulties that come with age, like hypertension and should visit antenatal care during pregnancy.
- ✓The government should facilitate infrastructures to teach and inform women, especially those residing in rural areas about the silent killer stillbirth that it is not because of an evil spirit called “Wukabi”.
- ✓Further studies should be conducted to identify other correlates of stillbirth that are not included and confirm the variables which are insignificant in this study because of many reasons and since regional variation are found significant spatial models can be applied to investigate spatial variations of experiencing stillbirth.

### Limitations of the study

Some of the limitations of the study are:-

- Since this study is based on secondary data from EDHS, 2011, we can study only the variables which are included in the questionnaire.
- Due to the presence of high missing values in 2011 EDHS data; some variables are not included in the study

## Abbreviations

ANC: Antenatal care
BMI: Body mass index
CSA: Central statistical agency of Ethiopia
DHS: Demographic and health survey
EDHS: Ethiopian demographic and health survey
IUGR: Intrauterine growth restriction
LMIC: Low and middle-income countries
LRT: Likelihood ratio rest
MDGs: Millennium development goals
MLEs: Maximum likelihood estimates
NCSS: The newborn cross- sectional study
PHC: Population and housing census
PMNCH: Partnership for maternal, newborn & child health
SNNPR: South Nation Nationalities and People Regional state
WHO: World Health Organization
